# Supplementation of Spring Pasture with Harvested Fodder Beet Bulb Alters Rumen Fermentation and Increases Risk of Subacute Ruminal Acidosis during Early Lactation

**DOI:** 10.3390/ani10081307

**Published:** 2020-07-30

**Authors:** Anita Fleming, Konagh Garrett, Kelly Froehlich, Matthew Beck, Racheal H. Bryant, Grant Edwards, Pablo Gregorini

**Affiliations:** Faculty of Agriculture and Life Sciences, PO Box 85084, Lincoln University, Lincoln 7647, Canterbury, New Zealand; konagh.garrett@lincolnuni.ac.nz (K.G.); kelly.froehlich@lincoln.ac.nz (K.F.); matt.beck@lincolnuni.ac.nz (M.B.); racheal.bryant@lincoln.ac.nz (R.H.B.); Grant.Edwards@lincoln.ac.nz (G.E.); Pablo.gregorini@lincoln.ac.nz (P.G.)

**Keywords:** fodder beet (FB), rumen function, grazing dairy cows, milk production, subacute ruminal acidosis, herbage intake, milk fatty acid composition

## Abstract

**Simple Summary:**

Fodder beet (FB) is widely used in grazing dairy systems of New Zealand to support early- and late-lactation milk production, however, the large fraction of water-soluble carbohydrate present in FB bulbs presents a risk of subacute and acute ruminal acidosis. Despite widespread use of FB across New Zealand, the incidence of ruminal acidosis using industry-recommended methods of feeding FB has not been investigated. This study analyzed the time-dependent changes to rumen fermentation, apparent dry matter intake, milk production, milk composition and plasma amino acid concentration of grazing dairy cows supplemented with moderate amounts (40% of dry matter intake) of FB during early lactation. Our findings indicate that incidence of subacute ruminal acidosis due to FB is greater than currently realized, as 25% of cows developed severe subacute ruminal acidosis following transition to target FB allocation (40% of daily intake). Across all cows, FB reduced rumen pH, feed conversion efficiency and was not advantageous to milk production. These results suggest methods for adapting cows to a diet containing FB require further evaluation to reduce the risk of subacute ruminal acidosis (SARA) experienced by individuals within the herd.

**Abstract:**

In a cross-over design, eight rumen cannulated dairy cows were used to explore the industry-recommended method for dietary transition to fodder beet (FB: *Beta vulgaris* L.) on changes to rumen fermentation and pH, milk production, dry matter intake (DMI) and the risk of subacute ruminal acidosis (SARA) during early lactation. Cows were split into two groups and individually allocated a ryegrass (*Lolium Perenne* L.) and white clover (*Trifolium repens* L.) diet (HO) or the same herbage supplemented with 6 kg DM/cow of harvested fodder beet bulbs (FBH). Dietary adaptation occurred over 20 days consisting of: stage 1: gradual transition to target FB intake (days 1–12, +0.5 kg DM of FB/d); stage 2: acclimatization (days 13–17) and stage 3: post-adaption sampling (days 18–20). Response variables were analyzed as a factorial arrangement of diet and stage of adaption using a combination of ANOVA and generalized linear mixed modelling. Dietary proportion of FB represented 22, (stage 1), 32 (stage 2) and 38% (stage 3) of daily DMI. One cow during each period developed SARA from FB and the duration of low pH increased with FBH compared to the HO treatment (*p* < 0.01). Rumen concentrations of lactic and butyric acid increased with FBH but concentrations of acetate, propionate and total volatile fatty acids (VFA) declined by 9.3% at day 20, compared to the HO treatment (*p* < 0.01). Treatments did not affect milk production but total DMI with supplemented cows increased during the final stage of adaptation and feed conversion efficiency (FCE kg milk/kg DM) declined with the FBH treatment. The occurrence of SARA in 25% of animals fed FB suggest it is a high-risk supplement to animal health and further evaluation of industry-recommended methods for feeding FB at the individual- and herd-scale are needed.

## 1. Introduction

New Zealand dairy farms have come to rely on fodder beet to increase body condition scores (BCS) within a short 6–8-week timeframe over the winter dry cow period. The popularity of fodder beet (FB) is driven by the potential to obtain large yields (>20 t dry matter: DM/ha) of high metabolizable energy bulbs (~12 MJ ME) that are ~90% utilized when grazed in situ [1,2,3]. The versatility to either graze in situ or harvest FB bulbs has led to systems which graze FB during late lactation and harvest residual winter FB to supplement the post-partum herbage supply in spring. Previous research suggests that there is limited advantage to milk production when FB is fed to grazing dairy cows [4,5,6], which may indicate that FB increases the substitution rate (kg DM herbage/kg DM FB) and reduces the milk response to the supplement (kg milk/kg DM supplement) when compared with alternative supplements such as pasture silage or cereal grains [7,8]. The low milk response to FB may also indicate impaired rumen function, as FB bulbs contain small amounts of fiber (<20%) and crude protein (CP; <10%) and large amounts of water-soluble carbohydrates (WSC; >65%) which are readily fermented within the rumen [9]. Thus, FB may limit the CP and fiber content of the diet and increase the risk of acute or subacute ruminal acidosis (SARA) [5,10,11]

Subacute ruminal acidosis is characterized by the accumulation of volatile fatty acids (VFA), which reduce rumen pH [12]. Low rumen pH may inhibit microbial activity and reduce digestibility of structural carbohydrates (cellulose and hemi-cellulose). Low rumen pH can also cause anorexia, reduced rumination and secretion of saliva which contains phosphate and bicarbonate buffers [13,14]. Exposure of the rumen wall to low pH conditions (pH < 5.6) impairs barrier function and can cause para-hyperkeratosis of epithelial cells, which limits animal production and welfare long-term [15,16]. The keratinization of the stratum corneum (outermost cells adjacent to ruminal contents) can reduce VFA absorption and bicarbonate exchange, which is a significant mechanism for neutralizing VFA and stabilizing pH (>50% of all VFA), and enhances the risk of SARA re-occurrence [17,18,19,20]. Reduced integrity of the barrier function can result in the translocation of endotoxins from the cell wall of Gram-negative bacteria into the portal circulation, causing inflammation-mediated liver changes and laminitis [14,21,22,23]. Other symptoms of SARA include reduced or erratic feed intake, milk fat depression and diarrhea [24]. Subacute ruminal acidosis is characterized by daily episodes of low pH [25] and reduced buffering capacity [12,25], but it is self-corrected. Declining pH proliferates *Lactobacilli* which produce lactic acid that is 10-fold the acidity of other organic acids and causes a downward spiral of rumen pH, leading to acute systemic acidosis that the animal is unable to correct [12].

Mean rumen pH has been identified as a poor technique for defining SARA in commercial dairy systems. While there is wide variation in response between individual animals, and limited clinical symptoms [24], Kleen and Cannizzo [26] described SARA from spot-samples of rumen fluid as severe when rumen pH is <5.5 and marginal when rumen pH is less than either 5.8 or 5.6, in cows on pasture-based diets. The use of other indicators, such as fecal consistency, low milk production and feed conversion efficiency (FCE), feed intake [27], increased circulation of endotoxins and acute phase proteins, provide some aid for determining the severity of SARA, but individually are poor indicators of the disease in grazing dairy cows [24,28,29]. Continuous monitoring of rumen pH is less practical for commercial dairy producers but is the most accurate approach for describing SARA [30]. The duration of pH below a threshold of 5.8 [31] for >180 min is defined as marginal SARA, while pH less than 5.6 for >180 min is defined as severe SARA [22], due to the relationship between pH and the impairment of microbial activity and structural integrity of the rumen epithelium.

Current industry recommendations suggest that non-lactating dairy cows can be safely transitioned to ad libitum (or appetite) amounts of FB over 14 days, by initially feeding ~2 kg DM/cow per day and increasing FB allocation by either 0.5 kg DM/cow every day or 1 kg DM/cow every second day [10,32]. While ad libitum intake of FB and high rumen pH > 6.0 have been reported for steers using the 14 d transitioning method [33], an animal’s risk of developing SARA is defined by its physiological state. Compared with beef steers, dairy cows may be more prone to SARA, with lactation and the stage of lactation increasing the level of risk [34]. For example, Waghorn, et al. [35] reported that five out of eight non-lactating dairy cows developed acute SARA using the 14 day transition technique among animals with a final allocation of over 80% of their diet as FB. Similarly, those authors observed acidosis in late-lactation cows which were being transitioned onto a diet of 45% and 60% FB [35]. Further analysis suggests that FB bulbs should not exceed 30–40% of daily DMI during lactation, due also to the low N content of the bulb [5,6,11]. Absorption of VFA across the rumen epithelium is a primary mechanism for maintaining rumen pH [36], but morphological changes to papillae size number increase gradually (6–8 weeks) post-partum and the absorptive capacity may be less than immediately required [34]. Thus, the definition of the upper limit that FB bulbs can be fed during early lactation, without compromising rumen pH or protein requirements, is needed.

The objective of this experiment was to measure the time-dependent changes to rumen function and fermentation. We hypothesized that early-lactation dairy cows can be safely transitioned and adapted to a moderate (~40% daily intake) intake of FB using industry approved methods.

## 2. Materials and Methods

An early-lactation grazing experiment was conducted between October and November of 2018 at the Lincoln University Research Dairy Farm (LURDF) Canterbury, New Zealand (43°38’ S, 172°27′ E). All procedures were approved by the Lincoln University Animal Ethics Committee (AEC 2018-22).

### 2.1. Experimental Design and Treatments

In a cross-over design, eight Holstein Friesian X Jersey dairy cows in their third lactation and fitted with a rumen cannula in February 2018, were separated into two groups based on days in milk (DIM; 29.63 ± 11.6, mean ± SD), milk yield (27.4 ± 5.25, kg/day) and liveweight (482 ± 50.0, kg). Cows were randomly assigned to one of two treatments: HO, an herbage-only control, consisting of approximately 19 kg DM/cow per day of an established perennial ryegrass and white clover sward that was above a post grazing height of 3.5 cm; or FBH, which consisted of 19 kg DM/cow per day of herbage and 6 kg DM of harvested FB bulbs (cultivar: Enermax). Response variables were analyzed as a 3 × 2 factorial arrangement of the diet and adaptation stages in a cross-over design consisting of two periods and eight replications per treatment. Each adaptation period consisted of three stages: transitioning during days 1–12 (stage 1), acclimatization during days 13–17 (stage 2) and full adaptation during days 18–20 (stage 3). Here transition refers to the gradual increase in FB allocation (+0.5 kg DM/cow/day) between days 1–12; adaption refers to the acclimatization of intake, VFA, rumen pH, milk fatty acids (reflect rumen biohydrogenation) and plasma amino acids, which were estimated to occur over five days (days 13–17), as previously reported for supplementation with concentrate [37]. Cows were assumed to be fully adapted and achieve consistent measures of production (estimated intake of DM and FB, milk production, milk composition and milk FA profiles) from the FBH diet between days 18 and 20. Dry matter intake and milk production data from day 20 were removed from the current analyses as cows were removed from the paddock at 2200 h and fasted overnight as part of a separate experiment. Cows were milked twice daily at approximately 0700 h and 1600 h and had free access to fresh water at all times except during milking. Following completion of the first period, a washout phase of 5 days occurred between periods to prevent first-order carry-over effects [38]. After the washout period, the same process which occurred in the first period was repeated, with cows provided the opposite dietary treatment.

### 2.2. Feed Management

Fodder beet was sown by precision drill on the 14th of November 2017 in a Templeton silt loam soil at 90,000 seeds/ha. Fodder beet was harvested, removed of residual leaf and transported to the experiment site prior to commencement of each period in order to maintain the chemical composition of the bulb. Fodder beet was allocated to each cow individually in plastic bins on a concrete feed pad following the morning milking. Cows remained on the feed pad for up to two hours or until completion of the FB meal before returning to a fresh allocation of herbage.

Following the morning milking, individual pasture breaks were allocated daily in horizontal strips across each paddock. Within the strip, each cow was individually separated using electric tape in order to estimate daily herbage intake of each animal. The botanical and chemical compositions of the sward were determined every three days prior to break allocation by collecting random hand grab samples of herbage (n = 5 per break) at grazing level (~3 cm above ground). On the same days, two random 0.2 m^2^ quadrats from each allocation were harvested to ground level following measurement of the compressed pasture height with a rising plate meter readings (RPM: Jenquip Ltd., Feilding, New Zealand). An additional two quadrats were taken from each allocation post-grazing. Harvested herbage was washed to remove soil contamination, dried at 60 °C in a force air oven to a constant weight to determine total DM. Dry matter yields were used to estimate herbage mass from the compressed sward height using multiple linear regression for both pre and post-grazing herbage mass in each period. The paddock, period and sward state (pre- and post-grazing) were significant (*p* < 0.05) and included in the final regression equations, while the effect of the treatment or cow was not significant and therefore was not included in the final model. The resulting regressions were used to determine herbage mass and estimate herbage dry matter intake from Equations (1)–(4):

Period 1: Post-grazing mass (kg DM/ha) = 1377.6 + (121.2 × RPM)(1)

Period 1: Pre-grazing mass = 1792.6 + (121.2 × RPM)(2)

Period 2: Post-grazing mass (kg DM/ha) = 391.1 + (121.2 × RPM)(3)

Period 2: Pre-grazing mass = 848.6 + (121.2 × RPM) *r^2^* = 0.755, *n* = 357, *p* < 0.0001. (4)

where rising plate meter (RPM) is the compressed pasture height measured in 0.5 cm increments.

To determine herbage allocation and apparent DMI approximately, 30 RPM readings were recorded in each allocation each day. Calibration equations were used to estimate pasture mass and apparent intake according to Equation (5).

Estimated DMI = (Pre-grazing mass − Post-grazing mass) × break size (ha)(5)

### 2.3. Plant Sub-Sampling and Analyses

Ryegrass was sampled for dry matter and nutritive value by random grab samples collected by hand at grazing height, which were bulked and separated into three sections to determine DM% (oven-dried at 60 °C for 48 h), chemical composition and botanical composition. Botanical components were sorted (perennial ryegrass, white clover, weeds) and oven-dried to calculate relative abundance in the sward. The third sample was frozen and stored until freeze-dried, ground and analyzed using near infrared spectrophotometry (NIRS. Model: FOSS NIRS Systems 5000, Maryland, USA).

Daily refusals of FB were collected and weighed to estimate daily FB intake. Three bulbs of FB were randomly selected from the face of the stack to analyze DM, chemical composition and fatty acid content, every third day. Fodder beet bulbs were quartered longitudinally, two quarters were selected randomly and minced separately using an electric hand blender. One sample was weighed and oven-dried (100 °C) for 72 h to determine DM%, and the second sample was frozen and stored freeze-dried, ground by a centrifugal mill (ZM200 Retsch GmbH; Haan, Germany) to pass through a 1 mm sieve, and then analyzed for chemical components (acid detergent fiber: ADF, neutral detergent fiber: NDF, organic matter: OM) using NIRS. Crude fat of FB and ryegrass was determined following [39]. Calibration equations for predicting WSC, CP, ADF, NDF and OM of FB were developed previously on samples of the FB bulbs. The R-squared values for CP, OM, WSC, NDF and ADF of both FB and ryegrass herbage were all above 0.9 and all samples were within the calibration range. Metabolizable energy content of both ryegrass and FB were calculated using the modified ADF (MADF) method; ME (MJ/kg DM) 14.55 − 0.015 × MADF [40].

### 2.4. Animal Samples and Analyses

Liveweight and milk yield (kg) were measured automatically at each milking (DeLaval Alpro Herd Management System, DeLaval, Tumba, Sweden) between days 0–19. To analyze milk fatty acid profiles, a representative sample of milk from each animal was obtained during milking using in-line milk meters. A sub-sample of this from each animal was composited by treatment from every third afternoon and morning milking between days 0–19 and was stored at −20 °C until analysis. Sub-samples from individual cows were collected on days 0 and 19 to determine milk urea N (MUN), proportion of protein, fat and lactose using Milkoscan (Foss Electric, Hillerod, Denmark, courtesy of Livestock Improvement Corporation, Christchurch, New Zealand). Fatty acid methyl esters of milk and plant material composited by plant (FB or herbage), diet and period, were prepared by trans-methylation and analyzed by gas chromatography (AOAC method 2012.13) (Shimadzu GC-2010, Japan with AOC-20i auto-sampler) using a Varian CP742 silica capillary column (0.25 × 100 m × 0.2 µm) courtesy of Fonterra Co-operative Group Ltd. A skimmed sample of milk was frozen at −20 °C until analyzed for milk urea nitrogen (MUN) by Randox RX Daytona analyses (Randox Laboratories, Ltd, Crumlin, UK). Daily feed conversion efficiency (FCE; kg whole milk/kg DMI) was calculated for milk yield by dividing by the estimated DMI.

Blood samples were collected in K_3_EDTA and Li heparin-coated vacuettes via the coccygeal vein or artery at approximately 16:00 on days 2, 11 and 20 of each period to measure concentrations of non-esterified fatty acid (NEFA) and free amino acids. Collected samples were immediately placed on ice until centrifuged at 3000× *g* for 15 min at 4 °C. Plasma was transferred to 2 mL Eppendorf tubes and stored at −20 °C until analysis. The concentration of NEFA in plasma was determined following Randox kit instructions. Concentrations of free amino acids in plasma were determined by high performance liquid chromatography (HPLC) using a 150 × 4.6 mm, C18 3u ACE-111-156 column (Winlab, Scotland), following the method of Heems, et al. [41].

Rumen pH was measured every 10 min using a wireless bolus (SmaXtec animal care GmbH, Austria). Boli were inserted into the rumen 7 days prior to the experiment and calibrated using a commercial buffer (pH 7.0), following manufacturer instructions. On three occasions of each period, random hand grab samples of rumen digesta were collected from the ventral sac of the rumen at 04:00, 08:00, 12:00, 16:00, 20:00 and 24:00 each day. Digesta was filtered through Chux cloth (Clorox, Australia) into two 2 mL microtubules to measure NH_3_ (acidified with 6 M sulfuric acid) and VFA concentration and were stored at −20 °C until analysis. Concentrations of VFAs were determined by gas chromatography using an SGE BP21 30 m × 530 µm × 1.0 µm wide-bore capillary column using an autosampler (AOC-20i) fitted to a Shimadzu GC-2010 gas chromatograph (Kyoto, Japan). Briefly, samples were thawed overnight at −4 °C and centrifuged at 13,000 rpm for 30 min at 4 °C (Beckman centrifuge JA20 rotor). An amount of 100 µL of supernatant was collected in a 2 mL Eppendorf tube, 20 µL of internal standard and 40 µL of metaphosphoric acid were added, and then the solution was vortexed for 30 min. Samples were diluted with acetone and water then vortexed again and passed through a 0.2 µm nylon syringe filter. Ammonia and L-lactate concentrations of the rumen fluid were determined enzymatically using Randox Daytona analyses following the kit instructions.

### 2.5. Statistical Analysis

Response variables were analyzed in R (r Core Team, 2018, v. 3.4.4.). Variables were analyzed as a factorial arrangement between diet and stage of adaptation. Apparent DMI, herbage intake (HI), FCE and NEFA, AA, milk FA and milk constituents of composited milk samples were analyzed by mixed effects ANOVA using the “lme” function of the lme4 package [42]. Treatment (i.e., diet), adaptation stage, the diet × adaptation stage interaction and period were fixed effects and individual cow was a random effect. Botanical components, fermentation products (VFA and NH_3_), yield of milk and milk constituents measured from individual cows (fat, protein, MS and lactose), and rumen pH (mean, min, max and duration of pH < 6.0, <5.8 and <5.6) were analyzed using the generalized linear mixed modelling function of the “lme4” package. Diet, adaptation stage, the diet × adaptation stage interaction, and period were fixed effects and cow was treated as a random effect. For repeated measurements within a day (VFA, NH_3_, and rumen pH), time was also included as a fixed effect, and interaction between time, diet and adaptation stage were also assessed. A number of rumen lactate samples were below the detectable limit and a zero-inflated generalized linear mixed model in the “glmmTMB” package was instead used to prevent over-dispersion [43]. Least square means were generated using the “emmeans” package [44] of R, and upon significance of the ANOVA, means were separated using pairwise contrasts. Differences were declared significant if *p* < 0.05 and tendencies were 0.05 < *p* < 0.1.

## 3. Results

One cow in the first period developed SARA (pH < 5.5 for 240 min per day) on day 10 of adaption, and her FB allocation was reduced to 3 kg DM, which was maintained until the end of the experiment. Another cow from the second period also developed SARA towards the end of adaptation (days 14–19) with a pH of <5.5 for between 110 to 190 min per day, thus, her allocation of FB was also reduced. Neither cow was removed from the experiment because pH was stabilized without intervention, which is a definitive characteristic of SARA.

### 3.1. Feed Measurements

While pre-grazing herbage mass was similar between treatments, cows supplemented with FB decreased utilization of pasture and displayed increasing post-grazing residuals compared with HO (*p* < 0.001, Table 1). Ryegrass accounted for over 90% of the biomass in the first period and 85% of the biomass in the second period (Table 1). The DM content of FB bulbs increased by 38.6% from period 1 to period 2, while DM content of herbage was similar for both diets. Proportions of ADF and NDF of the sward and in FB bulbs increased, while the ME content of herbage declined between periods. Based on random sampling of the grazed sward horizon, the herbage energy content was >11.0 MJ ME/kg DM and apparent ME content of FB bulbs exceeded 13.0 MJ ME/kg DM. Period did not alter the OM, WSC, CP or N content of herbage or FB. Herbage fatty acid content of FBH and HO diets and of FB bulbs are presented in Table 1. The content of FA in herbage was 83% greater than FB bulbs. The herbage grazed by the FBH treatment contained greater amounts of free fatty acids (14.2%) compared to herbage grazed by HO cows (Table 1).

### 3.2. Estimated Intake and Milk Production

There was no effect of diet on mean daily liveweight or mean seven-day liveweight. However, daily liveweight increased between stages one and two but remained consistent between stages two and three of adaptation (Table 2). The significant interaction of liveweight and period reflect a 2.2% decline from period 1 to period 2. Herbage intake was not restricted in either period as evidenced by low herbage utilization and high post grazing residuals, which exceeded target levels of 1550 kg DM/ha by between 30–100%. Intake of FB bulbs accounted for 22.2 (stage 1), 32.0 (stage 2) and 35.8% (stage 3) of daily DMI (Table 2). Utilization of FB was high during stage 1 of adaptation when allocation was less than 25% of the diet, but after allocation reached 5.5 kg DM/cow/d, utilization became more variable (Table 2). Across each stage of adaptation, cows refused 3.63%, (stage 1), 14.98% (stage 2) and 13.1% (stage 3) of FB bulbs offered (Table 2). Adaptation to FB bulbs caused substitution of herbage with FB bulbs by 0.57 ± 0.1 during stage one, 0.47 ± 0.14 in stage two and 0.31 ± 0.22 kg DM herbage/kg DM FB in stage three (mean ± SE). The effect of the adaptation stage or period was not significant, and a wide range of SE between days was observed (−2.70 to +8.57 kg DM/kg DM FB).

**Figure 1:**
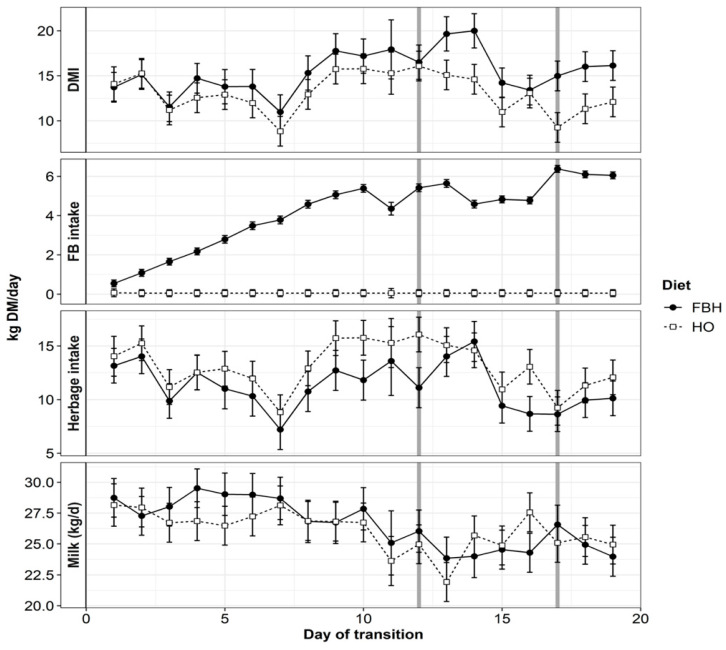
Apparent intake of total dry matter (DMI), herbage (HI), fodder beet bulbs (FB) and daily milk yield (kg) during dietary adaptation to either herbage-only (HO) or herbage and fodder beet (FBH) diets. Vertical reference lines represent the stages of FB adaptation where cows reached maximum allocation on day 12 and were fully adapted by day 18.

Apparent DMI was not affected by diet during stage one, but FBH increased DMI by 23.5% during stage two and by 25% during stage three of adaptation compared to HO (Table 2). Significant diet by day interactions were still apparent by day 19 of adaptation (Figure 1). Apparent herbage intake of cows fed HO were greater during stage one (*p* < 0.05), two (*p* = 0.075) and three (*p* < 0.05) of adaptation to the FBH diet. Despite greater estimated DMI, milk yield of cows fed FBH were similar to those fed HO between the second and third stages of adaptation (Table 2). Milk response (kg milk/kg DM of FB bulb) was not different between stages of adaptation or period, and averaged 0.46 ± 0.13 in stage one, 0.27 ± 0.18 in stage two and 0.39 ± 0.29 kg milk/kg DM FB during stage three and ranged from −2.13 to 3.76 kg milk/kg DM FB. Significant effects of adaptation stage were detected for milk solids response, which declined between stage one (0.13 kg MS/kg) and stage two (0.07 kg MS/kg), but was maintained in stage three of adaptation (0.07 kg MS/kg DM FB; *p* = 0.03). Milk solids response also declined between period one and period two (0.14 versus 0.03, kg MS/kg DM FB; *p* < 0.001) and ranged between −0.19 to + 0.77 kg MS/kg DM FB. 

Apparent DMI also varied between individuals and the stage of adaptation; the coefficient of variation of the FBH treatment was greater than HO at stage 3 of adaptation (21.7 versus 27.9%). Interaction of diet by stage or diet by day were not significant (*p* > 0.10) for milk constituents (fat, protein lactose) measured from composited milk samples, although FBH tended (*p* = 0.13) to reduce milk fat % during stage 1 of adaptation (Table 2). Milk fat (kg/d), lactose (kg/d), MS (kg/d) and total solids (kg/d) declined (*p* < 0.05) between stages one and two but increased (*p* < 0.05) between stages two and three of adaptation (Table 2). Supplementation of herbage with FB increased the fat % of bulk milk compared with HO but was not affected by the stage of adaptation. However, fat yield declined with stage and was reduced by FBH compared to HO (Table 2).

Milk composition from individual animals was not affected (*p* > 0.10) by treatment, other than lactose %, which declined (*p* < 0.05) with FBH (data not displayed). A similar relationship was identified in composited milk samples (Table 2), however, the effect of diet on lactose yield was not significant (*p* > 0.10). From composited milk samples, significant (*p* < 0.05) period effects were detected for fat (kg), protein (kg), kg MS, total solids, proportion of lactose and yield of whole milk (Table 3). Milk solid yield (kg/day) declined by (*p* < 0.05) 11.3% across all animals from day 1 to day 20 and mean solid yield also declined by 5.76% between periods (*p* < 0.01). However, significant dietary interactions of MS and percentage of MS were not detected (*p* > 0.10).

The FBH diet reduced FCE of milk compared to HO by 23%. There was no interaction between diet or adaptation stage, and no effect of stage on FCE (Table 2).

### 3.3. Milk Fatty Acids

There was minimal diet by day or diet by stage interaction for all fatty acids except for the sum of small chain fatty acids which were transiently reduced by the FBH diet during stage two and returned to HO levels by stage three of adaption (Table 3). The concentration of conjugated linoleic acid (CLA) declined with stage of adaptation (*p* < 0.05), but there was no significant effect of diet. Alternatively, the concentration of palmitic acid (C16:0) increased slightly between stage one and three, due to a tendency (*p* = 0.06) for cows fed FBH in stage one to produce greater concentrations of palmitic acid than those fed HO. Across all stages of adaptation, the FBH diet reduced proportions of long-chain and increased proportions of medium-chain fatty acids compared to HO (Table 3). The proportion of saturated fatty acids was increased (*p* < 0.05) by FBH, and saturated fatty acid content of milk tended (*p* = 0.06) to increase with stage (Table 3). Greater (*p* < 0.05) concentrations of lauric (C12:0), myristic (C14:0) and palmitic acids (C16:0) and biohydrogenation intermediates in milk from cows fed FBH increased the total medium and saturated FA content of milk (Table 3). Concentrations of branched chain and *trans* FA were not (*p* > 0.10) affected by diet or stage of adaptation, although their concentration tended to increase with adaptation stage (Table 3). The FBH diet increased the concentration of MUFA content of milk compared to HO, although the content of PUFA was not (*p* > 0.10) altered by diet (Table 3).

### 3.4. Rumen pH and VFA Patterns

**Figure 2:**
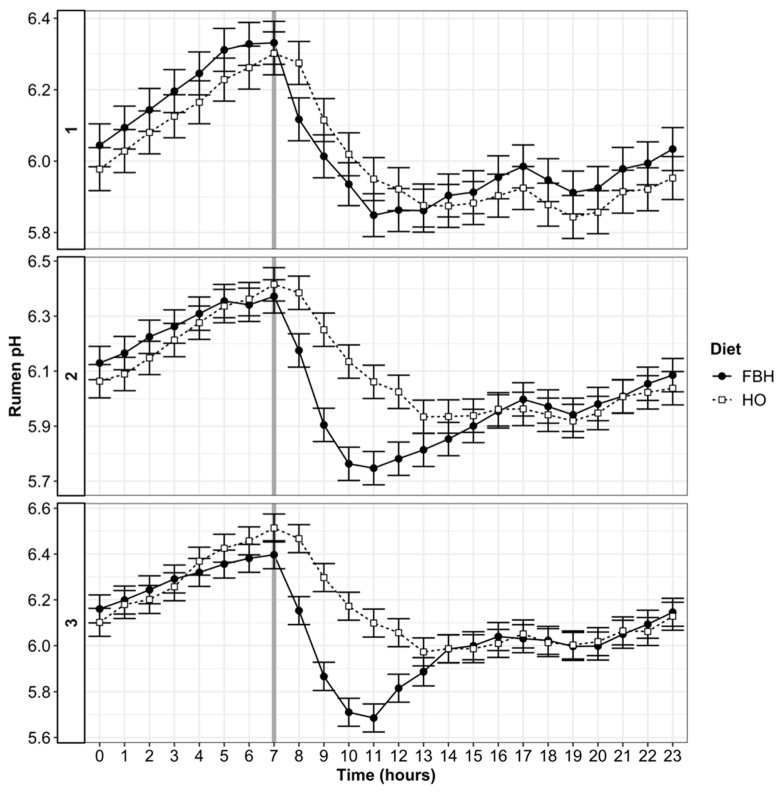
Diurnal pattern of rumen pH during dietary adaptation to herbage and fodder beet bulb (FBH) or herbage-only (HO) diets. Cows reached maximum FB allocation over 12 days (stage 1, top) and acclimatization by day 13 (stage 2, middle). Post-adaptation was assumed during days 18–19 (stage 3, bottom). Vertical reference lines represent the time that FB was fed, or fresh herbage was allocated.

Diet by adaptation stage interactions (*p* < 0.05) were detected for pH and duration of low pH (Table 4). The mean ruminal pH increased across all dietary treatments between stages one and three of adaptation (*p* < 0.01). During the first 12 days of adaptation, the mean rumen pH of cows fed FBH was greater (*p* < 0.05) than the pH of cows fed HO. However, the mean rumen pH of cows fed FBH declined (*p* < 0.05) compared to HO during stages two and three (Table 4). Significant diet effects on maximum pH were not apparent (*p* > 0.10), although max pH increased (*p* < 0.05) with stage of adaptation. The FBH diet reduced (*p* < 0.05) the daily nadir of pH by 4.6% during stage two and 4.0% during stage three, compared with HO. The gradual increase in FB allocation during stage one caused the duration where pH was < 6.0 and < 5.8 to increase (*p* < 0.05) by 41 and 16 min/d, respectively, when compared with cows fed HO (Table 4). However, the duration where pH was < 5.6 was also 8.7 min longer (*p* < 0.001) for cows fed FBH than those fed HO during stage one of adaptation (Table 4). The FBH diet increased (*p* < 0.05) in the duration where pH was < 6.0 by 49 min/d, in the duration where pH was < 5.8 by 16 min/d and in the duration where pH was < 5.6 by 19.1 min/d compared with cows fed HO during stage two of adaptation (Table 4). During the third stage of adaptation, the duration where pH was less than 6.0 (20 min), 5.8 (9 min) and 5.6 (9 min) was greater (*p* < 0.05) for cows fed FBH than for those fed HO (Table 4). The duration of low pH during stage three declined (*p* < 0.05) with both diets compared to stage two of adaption. Analysis of diurnal rumen pH indicated significant (*p* < 0.05) diet by stage by hour interactions of rumen pH (Figure 2). During the first stage of adaptation, rumen pH of both diets declined following allocation of herbage or FB after the morning milking. During stage two, pH patterns were divergent by diet, as following the FB meal, pH declined to a nadir of 5.5 and remained below 5.6 until 14:00 while the daily nadir of HO animals averaged 5.7 and pH from both groups remained below 5.8 until after midnight. During the third stage of adaptation, nadir rumen pH in cows fed FBH reached 5.5 at 10:00 (two hours post-FB allocation), pH was >5.6 by 13:00, while nadir pH of 5.7 was maintained for the HO diet (Figure 2).

Significant (*p* < 0.05) diet by adaptation stage interactions and diet by time by stage interactions were detected for all rumen fermentation products except the A:*p* ratio (Table 5). From all samples measured for lactic acid, 45% were above the detectable limit (>0.00001 mmol/L) and generally were detected between 12:00 and 16:00. The concentration of lactic acid was greater (*p* < 0.05) for cows fed FBH and increased on day 11 and declined again by day 20 of adaptation (Table 5). Day of adaptation to the FBH diet had a significant effect (*p* < 0.05) on all VFA and differences between the treatments were observed from the 11th day of adaptation to FB (Table 5). The A:*p* ratio tended (*p* = 0.06) to increase with the FBH diet, although differences were small (3.66 versus 3.65). Fodder beet reduced the concentration of propionate by 16% (stage 2) and 12% (stage 3) compared to the HO treatment. The concentration of acetate also declined by 21% (stage 2) and 20% (stage 3) when FBH was fed when compared to HO counterparts. The FBH diet increased the concentration of butyrate by 16% during stage 2 and 22% during stage 3 (Table 5), however, total VFA concentrations declined by 13% (stage 2) and 10% compared to cows fed HO (Table 5).

**Figure 3:**
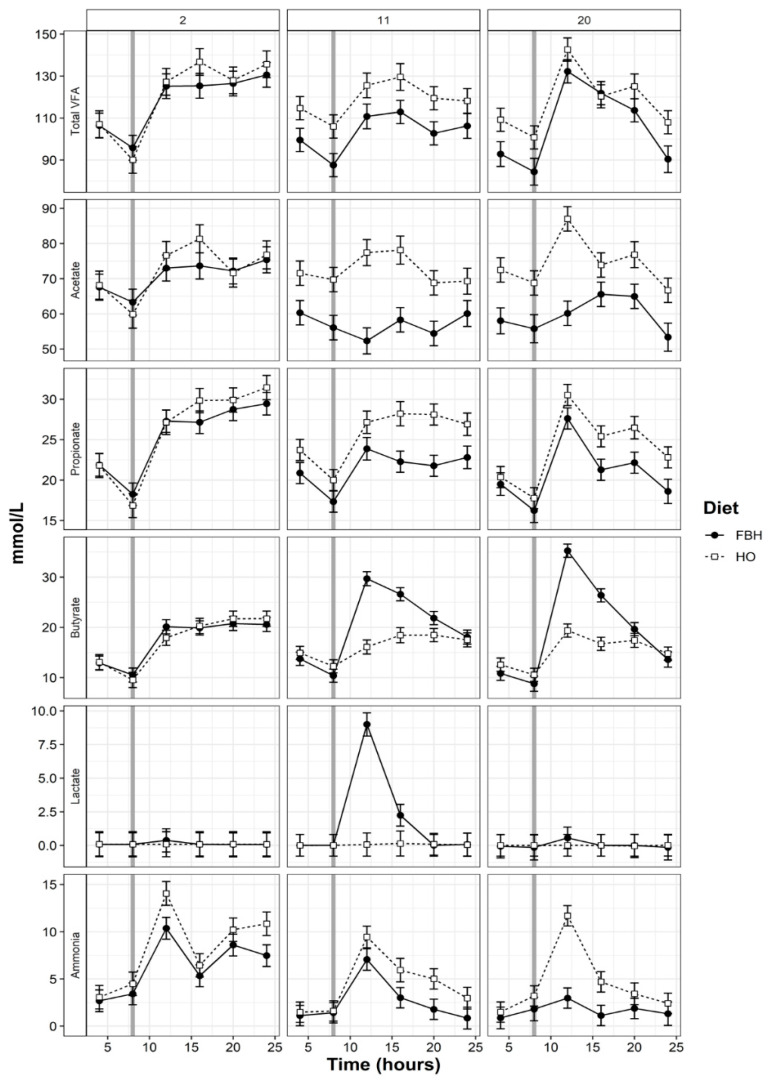
Diurnal fluctuation of fermentation-end products and total volatile fatty acid concentrations of rumen fluid collected from cows during adaptation (days 2, 11 and 20) to either herbage and fodder beet bulb (FBH) or herbage-only (HO) diets. Vertical reference lines represent the time of FB meal or fresh herbage allocation.

**Figure 4:**
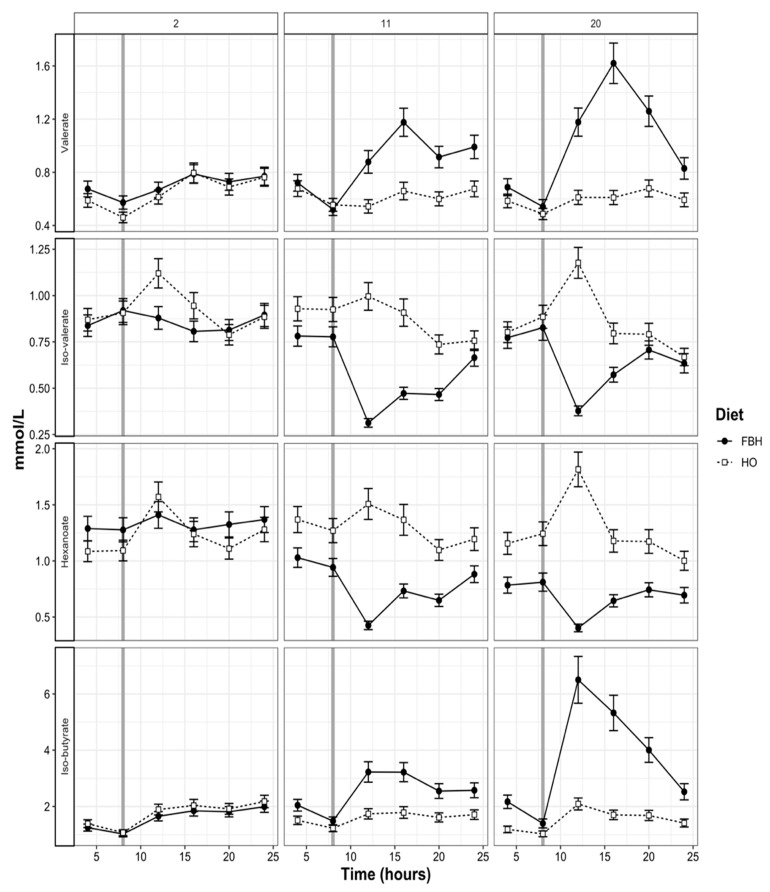
Diurnal fluctuation of valerate, iso-valerate, hexanoate and iso-butyrate concentrations of rumen fluid collected from cows during adaptation (days 2, 11 and 20) to either herbage and fodder beet bulb (FBH) or herbage-only (HO) diets. Vertical reference lines represent the time of FB meal or fresh herbage allocation.

Following consumption of FB bulbs in the morning, concentrations of butyrate and propionate increased, spiking around midday. The concentration of acetate from cows fed FBH remained constant between 50–60 mmol/L on day 11 of adaptation, while concentrations ranged between 80 and 60 mmol/L for cows fed HO. On day 20, concentrations of acetate from cows fed FBH peaked at 70 mmol/L at 15:00, plateaued during the early evening and declined just prior to midnight. In comparison, the concentration of acetate from cows fed HO spiked at 90 mmol/L at midday and declined gradually over the following 12 h (Figure 3). Total VFA and ammonia concentrations peaked across both dietary treatments between 08:00 and 12:00 and declined slightly thereafter. The FBH diet increased concentrations of iso-butyrate by 69.2% at midday compared to HO concentrations on day 20 of adaptation (Figure 4). Concentrations of valerate and hexanoate were increased (*p* < 0.05) by FBH compared to HO, while concentrations of iso-valerate declined (*p* < 0.05) with FBH (Table 5). Dietary differences between valerate, iso-valerate and hexanoate also increased (*p* < 0.05) with stage of adaptation (Table 5; Figure 4). Differences in lactate, propionate, total VFA and hexanoate concentrations between periods were significant (*p* < 0.01).

### 3.5. Plasma Amino Acids

The concentrations of blood metabolites were largely unaffected by diet and the interaction of diet with the adaptation stage was only significant for glycine and serine concentrations (Table 6). The glycine concentration of cows fed FBH was 130% greater (*p* < 0.05) than those fed HO at day 11 of adaptation and 28% greater (*p* < 0.05) at day 20. Serine concentrations declined (*p* < 0.05) in cows fed FBH during day 20 (15.2%) compared to cows fed HO. Significant diet effects were also detected for taurine, and isoleucine (Table 6), which were all increased (*p* < 0.05) by the FBH diet. The effect of adaptation stage was also significant (*p* < 0.05) for NEFA, as concentrations declined across both dietary treatments between days 2, 11 and 20 of adaptation. Across the entire adaptation period, cows fed HO on average had slightly but significantly elevated NEFA compared with cows fed FBH (*p <* 0.05).

## 4. Discussion

This experiment examined the time-dependent changes to rumen function and fermentation as animals adapted to a diet of herbage and FB bulbs using current industry recommendations for transitioning [10,32]. There were no interactions between diet and stage for bio-hydrogenation intermediates in milk or amino acids (except serine and glycine) in plasma. Such lack of interaction may suggest initial adaptation to the FBH diet, although diet by adaptation stage interactions were still apparent by day 19 and 20 for apparent DMI, FB intake, total VFA and rumen pH. The occurrence of SARA in two individuals (one during each period) indicates that the null hypothesis should be rejected, as use of industry recommended transitioning methods did not prevent SARA in individual animals. The following discussion focuses on whether cows fed the FBH diet had fully adapted and some factors which may have prevented individual cows from achieving consistent FB intake and rumen pH.

### 4.1. Rumen pH and SARA

Consumption of FB bulbs during the second and third stage of adaptation caused sub-optimal pH of all cows fed the FBH diet. A duration of pH < 5.6 represent the time frame in which fibrolytic activity of the rumen is impaired [45]. Cows fed the FBH diet consistently experienced longer episodes of pH < 5.6 compared to animals fed HO (Table 4). By day 15 of adaptation, the time that pH was less than 5.6 spiked at 45 min/day, although intake of FB was consistent at 4.5 kg DM/cow during this time (Figure 1). While the FBH diet increased the duration of pH < 5.6 compared to HO, the duration that cows experienced low pH was less than that reported in the study of Krajcarski-Hunt, et al. [45], in which SARA was deliberately induced (45 versus 594.4 min/day). When pH duration was averaged across all cows, the FBH diet did not cause widespread SARA as defined by Gozho, et al. [46] as pH < 5.6 for > 3 h. However, Zebeli, et al. [16] found circulating concentrations of the pro-inflammatory serum albumin A increased by 0.21 mg/L each minute that rumen pH was < 6.0. Circulating concentrations of tryptophan and histidine may be linked to concentrations of serum albumin [47]. However, FB did not alter plasma concentrations of either histidine or tryptophan (Table 6). The increased duration of low pH caused by the FBH diet may enhance liver-mediated inflammation, laminitis, mastitis, metritis ruminitis and oxidative stress in some individuals within the herd [22,25,48,49], and further evaluation of serum albumin A concentration is required.

Rumen pH was reduced following consumption of FB in the morning; however, rumen pH of cows fed the HO diet was also low and variable (Table 4). Both FBH and HO diets resulted in rumen pH that was lower than previously reported for housed cows fed increasing proportions of FB and herbage [6]. The continuous measurement of rumen pH in the current experiment should be more accurate and warrant more regular measurements than every 2 h as practiced in [6,30]. Zebeli, et al. [16] and Mertens [50] suggest that the duration of low rumen pH also increases when the NDF and physically effective NDF (i.e., stimulates chewing and secretion of saliva) content of herbage is less than 42%. In comparison, the NDF content of herbage fed in the current experiment was between 36–41%, while NDF of the FB bulbs was comparably less (13–14% NDF) and probably accounted for the generally low pH observed and further decline in the FBH treatment. High-nutritive value herbage is readily fermentable and contains minimal amounts of NDF or ADF and incidence of SARA is probably underestimated in grazing dairy cows [24]. Thus, the low NDF and high digestibility of FB bulbs does not appear to complement the low NDF content of herbage, particularly post-partum when the risk of SARA is elevated [34,51].

Daily DMI of cows fed FBH was variable (Figure 1) and the CV of DMI observed for cows fed FBH was still greater than those fed HO at stage 3 of adaptation (28% and 21%, respectively). While low DMI is generally understood to be the primary symptom of severe SARA, the variation of DMI between days is indicative of mild SARA [52] and Gozho, et al. [46] reported that DMI increased transiently from 0.8 to 1.0 kg DM during starch-induced SARA. Variation of DMI reflects the cyclical accumulation of VFA within the rumen and their potent anorexigenic control on voluntary intake [14,53,54]. In the current experiment, daily DMI of cows fed FBH was also cyclic, increasing on day 13 of adaptation to 20 kg DM/cow and declining to 5 kg DM/cow on day 14. The reduction in DMI from cows fed HO on day 13 may indicate that ruminal sampling on day 11 reduced the time available for grazing. However, disrupted grazing time does not completely account for the observed variation in intake between treatments or the changes observed between days 13 and 14 (Figure 1). It should be noted, however, that DMI was also variable across the HO treatment (Figure 1) and, despite the limited advantage of FB to milk production, FCE may have been confounded in the current experiment due to greater estimated DMI of cows fed FBH.

The high FCE experienced by cows fed HO may indicate that cows consumed insufficient DMI to meet energy demands for lactation. The cows in this study were producing 24.5 kg of milk per day which, based on AFRC (Agricultural and Food Research Council) formula for a 490 kg cow, will have an ME requirement of approximately 200 MJ ME/day (equivalent to 16–17 kg DM/day). The FCE of cows fed HO during stage three was 2.1 (HO) kg milk/kg DM, which is greater than the FCE of 1.8 kg milk/kg DMI reported by [55], and FCE of cows fed FBH in the current experiment was less than the published results (1.61 kg milk/kg DMI). High FCE was due to similar milk production as cows fed FBH, despite low DMI. While residual post-grazing herbage cover of cows fed HO suggests that herbage allocation was not limiting (Table 1), low DMI of the HO treatment may have increased mobilization of adipose tissues and diluted the energy used for maintenance [56]. The increased circulation of NEFA observed in cows fed HO may indicate a greater energy deficit when compared to cows fed FBH. However, increased circulation of NEFA is associated with an increased concentration of C18:1 *cis* 9 in milk [57,58], although this was not observed in cows fed HO (Table 3). Therefore, the increased circulation of NEFA in cows fed HO while statistically significant is probably not biologically meaningful.

### 4.2. Patterns of Rumen Fermentation

The 23% reduction in FCE in the FBH treatment may alternatively suggest reduced digestion efficiency either due to greater DMI and rumen passage rate [59] or low rumen pH [25]. While the FBH diet also increased DMI by 25%, this should not have caused a near equal decline in FCE. Auldist, et al. [60] supplemented an herbage-only diet with low (2.5 kg DM/day) or high (5.0 kg DM/day) amounts of grain during early lactation (60 DIM) and reported that low and high grain allocation increased DMI by 12% and 20%, respectively, and did not alter FCE compared to cows fed herbage only. The reduced FCE caused by feeding FB is abnormal compared to alternative supplements, which may suggest additional factors such as the reduction in rumen pH reduced digestion efficiency, which ultimately limited the marginal milk response to the supplement. Circulating serine concentrations also declined by 15% in cows fed FBH on day 20 of adaptation. Serine concentrations of all cows were low compared to a previous study of cows fed a high-energy total mixed ration (75 versus 43 μmol/L) [61]. The reduction in serine in cerebrospinal fluid [61] and in plasma [62] of lactating dairy cows fed a restricted diet indicates that serine is an anorexigenic signal, which may also explain the increased DMI of the FBH treatment. However further research regarding the relationships between plasma concentrations of serine, supplementation of FB, the effect of SARA and DMI are needed.

Diurnal patterns of all fermentation end-products reflected the time of feeding (FB or herbage) in the morning (Figure 3, Figure 4). Despite greater apparent DMI, cows fed FBH experienced lower concentrations of total VFA, as both concentrations of acetate and propionate declined when compared to cows fed HO. Although Pacheco, et al. [6] also reported a lower concentration of acetate from cows when FB was fed as 23% or 45% of DMI, differences were not as large as those observed in the current study. Reduction in total VFA concentration has only been reported for non-lactating dairy cows fed large amounts of FB (>60% DMI) [6]. While the greater concentration of butyrate experienced by cows fed FBH has been previously observed in vivo and in vitro, the decline in propionate has not been reported for grazing dairy cows fed FB and herbage [6]. Daily mean butyrate concentrations observed from cows fed FBH were within a similar range (15–17 mmol/L) to a previous study, but butyrate concentration from cows fed HO were slightly greater than those observed by Pacheco, et al. [6] (14.3–15.2 versus 10 mmol/L). While concentrations of propionate did increase following the consumption of FB bulbs, cows fed HO experienced a greater spike of propionate following allocation of herbage in the morning, reflecting the high digestibility of spring pasture (Figure 3). The concentration of VFA provides an indication of microbial activity, but may be confounded by the rate of VFA removal from the rumen, resulting in the underestimation of total VFA concentrations [63]. Cows fed FBH consumed 12.9% less herbage during stage 3, which does not account for the 26.1% decline in mean acetate concentrations observed on day 20 when compared to the HO treatment. The decline in total rumen VFA, FCE and plasma serine concentration and further support the conclusion that FBH impaired rumen function.

The spike of lactic acid concentration coincided with the daily nadir of rumen pH, following intake of FB in the morning. A significant effect of an individual cow was detected for lactic acid concentrations; however, surprisingly, cows that experienced the greatest lactic acid concentrations were not always the same animals that developed SARA, indicating individual resilience to low rumen pH. Unfortunately, D-lactate was not detectable using GC methods, and L-lactate was measured enzymatically instead. While L-lactate found in the rumen is the same form produced from glucose metabolism in muscle, D-lactate is entirely of microbial origin and is not formed by mammalian cells [64]. However, the elevated concentration of L-lactate from animals fed FBH still indicates altered microbial community profiles, although the proportion of D-lactate is expected to increase and L-lactate to decline at low rumen pH [14]. The increase in lactate observed in cows fed FBH was minor compared to previous SARA challenges [65] but may have favored the formation of butyrate due to the increased concentration of H^+^, osmotic pressure and the altered metabolism of the rumen epithelia [12,66,67].

Lactic acid represented a minor fraction of VFA and does not account for the reduction in total VFA concentration of cows fed FBH. Therefore, low rumen pH was likely influenced by both the rate of VFA accumulation and the concertation of lactate in the rumen. Butyrate is a favored energy substrate for epithelial cells and may indicate altered epithelial metabolism [68,69,70,71]. Intra-ruminal administration of butyrate caused a 4-fold proliferation of epithelial cells [72]. Specifically, cell thickness of the stratum corneum increased by 15 cells, which is the fourth cornified layer of the rumen epithelia [73] and is linked to an increase in localized inflammation, ruminitis and parakeratosis [68,74]. Like propionate, intravenous administration of butyrate causes hyperglycemia, but has also been found to induce a rapid and sustained increase in serum insulin in sheep [75]. Increased sensitivity to insulin may also explain the lower circulatory concentration of NEFA observed from cows fed FBH, which contrasted to the decline in lactose content that has also been observed previously in cows fed FB [4] and has been identified in cows induced with SARA using barley grain [49]. Lactose is comprised of a glucose and a galactose residue, which are both formed from hepatic oxidation of propionate [76] and which declined when FB bulbs were fed (Table 2). The majority of butyrate formed in ruminal fermentation is used by the rumen epithelia [12,66], and it is not clear whether low rumen pH conditions and increased concentration of butyrate may have increased permeability of the rumen epithelia, altering insulin signaling or epithelial ketogenesis [77,78]. While the FBH diet appeared to improve post-partum energy status, the reduction in circulating NEFA and percentage of lactose in milk reflect altered fermentation dynamics and reduced substrate availability for hepatic gluconeogenesis.

### 4.3. Rumen Adaptation

Of further interest to the time-dependent adaptation of dairy cows fed FB is the absorptive capacity of the rumen wall. Passive diffusion of undissociated VFA (HVFA), and alkalization and buffering of VFA by phosphate and bicarbonate (HCO_3_^−^) in saliva, are mechanisms which help to stabilize rumen pH [12]. However, HCO_3_^−^ mediated transfer of dissociated VFA (VFA^+^) across the epithelium removes >50% of all hydrogen ions from the rumen and is a primary mechanism for maintaining rumen pH [20,36,79]. Both structural (proliferation of epithelial cells, increased papillae size) and width and metabolic adaptations (expression of genes which regulate VFA transport pathways) can improve absorption of VFA by 300% [63,80,81,82]. While morphological changes occur over an extended time frame (16 days–several weeks) post-partum [80,81], Etschmann, et al. [82] reported that 70% of metabolic changes to the rumen epithelium occurred within the first 7 days following a change of diet. Therefore, the 12-day transitioning, and 5-day adaption period used in the current experiment should have been sufficient for metabolic development of the rumen epithelium. However, rumen pH still declined with the FBH diet and transitioning did not prevent SARA in two out of eight cows.

The inability to maintain intake of herbage or FB bulbs using individualized feeding methods is concerning for commercial dairy producers which feed FB during lactation due to the limited clinical symptoms and the apparent individual risk of SARA within the herd. The individualized response to FB feeding and susceptibility to SARA have also been identified in cows fed a starch-rich diet [18,83,84,85]. Both the rate of VFA accumulation and rate of VFA absorption are factors associated with the individualized response to a SARA challenge, although the specific mechanisms involved in the response are not yet clear [83]. In commercial dairy systems, group feeding of FB may further enhance the risk of SARA due to both to the increased intake rate caused by competition [86] and reduced herbage allocation, as FB is often used to mitigate herbage deficits, as noted by Fleming et al. (in press). Further information regarding individual susceptibility to ruminal acidosis over an extended period of time is needed to develop FB feeding methods that prevent SARA in cows which are predisposed to this disease.

## 5. Conclusions

Dairy cows fed moderate allocations of a high sucrose supplement such as FB have an increased duration of pH < 5.6. The FBH diet reduced FCE, rumen VFA concentrations and plasma serine concentrations, indicating impaired fibrolytic activity of the rumen, even after 20 days following industry-recommended methods. Consequently, industry guidelines for feeding FB to early-lactation dairy cows can increase animal health risks and reduce animal performance. Further evaluation of industry guidelines for the duration and methods of FB transitioning, adaptation and upper limits of daily intake during early lactation are needed.

## Figures and Tables

**Table 1:** Pre- and post-grazing pasture mass, botanical composition (%) of sward and chemical composition^2^ (%) including free fatty acids (mg/g dry matter (DM)) of herbage and fodder beet root (FB) fed as an herbage-only diet (HO) or a herbage + FB diet (FBH). *p* < 0.05 *; *p* < 0.01**; *p* < 0.001 ***.

Diets	Period 1			SE ^1^	Period 2			SE	*p*-Value	
	Herbage		FB Bulb		Herbage		FB Bulb		Diet	Period
	HO	FBH	FBH		HO	FBH	FBH			
Pre-grazing (kg DM/ha)	5497	5453	-	86	3478	3596	-	61	0.14	***
Post-grazing (kg DM/ha)	2823	3050	-	68	1953	2277	-	59	***	***
Area (m^2^/cow/day)	53.6	52.2	-	1.06	76.3	73.4	-	1.71	0.18	***
Allocation (kg DM/cow)	26.4	26.0	-	0.38	29.1	28.7	-	0.45	0.52	***
Pasture botanical										
Ryegrass % DM	91.6	95.2	-	2.04	86.4	85.6	-	1.96	0.31	0.26
White clover % DM	1.36	1.14	-	1.67	5.02	6.14	-	2.08	0.71	0.13
Weeds % DM	3.96	3.73	-	2.83	8.74	10.6	-	3.39	0.62	0.06
Dead % DM	5.27 ^a^	2.89	-	0.707	1.52 ^b^	1.95	-	0.914	0.38	0.14
Chemical composition										
DM % DM	14.7 ^c^	14.2 ^c^	12.7 ^a^	0.36	17.5 ^d^	18.1 ^d^	20.7 ^b^	0.60	0.39	***
OM % DM	91.5 ^b^	91.4 ^b^	94.2 ^a^	0.26	91.8 ^b^	91.7 ^b^	93.7 ^a^	0.29	0.66	0.75
ADF % DM	21.0 ^c^	21.2 ^c^	7.81 ^a^	0.123	23.3 ^d^	23.6 ^d^	8.15 ^b^	0.130	0.12	***
NDF % DM	36.6 ^d^	37.7 ^c^	13.0 ^a^	0.185	41.7 ^e^	41.8 ^e^	14.0 ^b^	0.241	0.07	***
WSC % DM	21.1 ^b^	20.6 ^b^	63.9 ^a^	0.39	20.5 ^b^	20.2 ^b^	59.4 ^a^	0.41	0.36	0.15
CP % DM	15.6 ^b^	16.0 ^b^	8.23 ^a^	0.27	15.7 ^b^	15.5 ^b^	9.39 ^a^	0.37	0.69	0.29
ME (MJ/kg DM)	11.2 ^a^	11.2 ^a^	13.4 ^b^	0.03	11.1 ^a^	11.0 ^a^	13.2 ^b^	0.03	***	***
Fat % DM	2.30 ^c^	2.72 ^b^	0.59 ^a^	0.084	2.12 ^c^	2.54 ^b^	0.40 ^a^	0.088	***	0.13
∑SFA (mg/g DM)	4.32 ^b^	4.49 ^b^	0.66 ^a^	0.077	4.27 ^a^	4.60 ^a^	0.76 ^b^	0.077	***	0.07
∑MUFA (mg/g DM)	1.04 ^d^	1.17 ^f^	0.43 ^b^	0.024	0.95b ^c^	1.09 ^e^	0.34 ^a^	0.0243	***	***
∑PUFA (mg/g DM)	9.97 ^e^	13.21 ^f^	1.28 ^b^	0.100	9.07 ^c^	9.76 ^d^	0.85 ^a^	0.100	***	***
∑Total FA (mg/g DM)	18.9 ^d^	21.6 ^f^	3.29 ^b^	0.47	16.8 ^c^	19.5 ^e^	1.19 ^a^	0.47	***	***

^a–e^ Means within rows with different superscripts are significantly different (*p* < 0.05). *p* < 0.001 ***. ∑SFA: sum of saturated fatty acids; ∑MUFA: sum of mono-unsaturated fatty acids; ∑PUFA: sum of poly-unsaturated fatty acids; ∑Total FA: sum of all fatty acids. ^1^ SE: standard error of estimated marginal means. ^2^ ADF: acid detergent fiber, NDF: neutral detergent fiber, WSC: water soluble carbohydrate, OM: organic matter, CP: crude protein, N: nitrogen, ME: metabolizable energy.

**Table 2:** Animal liveweight (kg) daily yield of milk and milk solids (MS), estimated daily allocation (HA; kg DM/cow) and apparent intake of herbage (HI), fodder beet (FB) and total dry matter (DMI) during adaptation to either grazed herbage + FB (FBH) or the herbage-only diet (HO).

Adaptation Stage ^1^	Stage One		SE ^2^	Stage Two		SE	Stage Three		SE	*p*-Value			
Diet	HO	FBH		HO	FBH		HO	FBH		Diet	Period	Stage	D × S ^5^
Liveweight (kg)	478 ^a^	480 ^a^	4.2	487 ^a^	488 ^a^	5.3	486 ^a^	484 ^a^	6.4	0.45	***	**	0.87
7d LWT ^3^ (kg)	493 ^a^	494 ^a^	3.1	496 ^a^	498 ^a^	3.3	497 ^a^	499 ^a^	3.5	0.40	***	**	0.78
DMI ^4^ (kg DM/day)	13.8 ^a^	14.5 ^b^	0.31	11.6 ^c^	15.3 ^b^	0.42	11.6 ^c^	15.8 ^d^	0.61	***	***	0.55	0.07
HI (kg DM/day)	13.9 ^b^	11.3 ^a^	0.39	11.6 ^c^	10.5 ^c^	0.51	11.6 ^c^	10.1 ^c^	0.747	***	**	*	0.64
FB (kg DM/day)	0 ^b^	3.2 ^a^	0.12	0 ^b^	4.9 ^c^	0.21	0 ^b^	5.7 ^d^	0.31		**	***	
FB utilization %		94.4	2.22		80.0	2.12		82.7	1.72		0.52	***	
HA (kg DM/d)	18.5 ^a^	17.8 ^a^	0.28	16.6 ^b^	16.4 ^b^	0.40	15.6 ^b^	16.5 ^b^	0.61	0.27	0.38	***	0.28
Fat (%)	5.09	4.57	0.155	4.47	4.55	0.220	4.46	4.59	0.311	0.13	0.19	0.57	0.20
Fat (kg)	1.29 ^a^	1.21 ^a^	0.034	1.06 ^b^	1.06 ^b^	0.049	1.10 ^b^	1.08 ^b^	0.067	0.22	***	0.11	0.51
Protein (%)	3.68	3.76	0.053	3.79	3.67	0.075	3.55	3.71	0.106	0.08	0.50	0.52	0.92
Protein (kg)	0.94 ^a^	1.00 ^a^	0.026	0.88 ^b^	0.88 ^b^	0.036	0.86 ^b^	0.89 ^b^	0.052	0.16	*	*	0.68
Lactose (%)	5.12 ^a^	5.05 ^b^	0.023	5.14 ^a^	5.08 ^b^	0.033	5.08 ^b^	5.04 ^b^	0.047	*	0.48	**	0.88
Lactose (kg)	1.30 ^a^	1.34 ^a^	0.029	1.23 ^b^	1.18 ^b^	0.041	1.23 ^b^	1.21 ^b^	0.058	0.89	**	**	0.53
MS (%)	8.87	8.43	0.158	8.26	8.42	0.224	8.09	8.38	0.316	0.33	0.15	0.52	0.18
MS (kg)	2.23 ^a^	2.20 ^a^	0.046	1.94 ^b^	1.94 ^b^	0.066	1.94 ^a^	1.99 ^a^	0.093	0.88	***	*	0.87
Milk (kg)	25.4 ^a^	26.5 ^a^	0.56	23.9 ^b^	23.3 ^b^	0.80	24.2 ^a^	24.0 ^a^	1.13	0.51	**	**	0.46
MUN ^6^	5.3 ^a^	7.3 ^a^	1.09	8.2 ^ab^	4.2^a^	1.52	4.6 ^a^	4.0 ^a^	2.15	0.93	0.49	0.90	0.09
FCE (kg milk/kg DMI)	2.00 ^b^	1.78 ^ab^	0.149	2.07 ^b^	1.55 ^a^	0.154	2.10 ^b^	1.61 ^a^	0.190	***	0.60	***	*

^a–e^ means in the same row with different superscripts differ (*p* < 0.05). *p* < 0.05 *; *p* < 0.01**; *p* < 0.001 ***. ^1^ Stage one: days 1–12; stage two: days 13–17; stage three: days 18–20. ^2^ SE: standard error of estimated marginal means. ^3^ Average liveweight over 7 days. ^4^ DMI was estimated based on estimated DMI = (pre-grazing − post-grazing mass) × break size (ha). ^5^ Diet by stage interaction. ^6^ milk urea nitrogen.

**Table 3:** Change of total small (<C10), medium (<C16 and >C10) and long chain (>C16) fatty acids of milk during dietary adaptation to either herbage-only (HO) or herbage + fodder beet bulb (FBH) diets.

Adaptation Stage ^1^	Stage 1		SE ^2^	Stage 2		SE	Stage 3		SE	*p*-Value			
	HO	FBH		HO	FBH		HO	FBH		Diet	Stage	Period	D × S ^4^
g/100g of FA													
Σ Small chain	7.26 ^a^	7.41 ^a^	0.108	7.42 ^a^	6.81 ^b^	0.153	7.26 ^a^	7.23 ^a^	0.216	0.37	0.22	0.11	*
Σ Long chain	32.8 ^b^	28.2 ^a^	1.47	32.4 ^b^	28.0 ^a^	2.07	26.4 ^a^	23.5 ^a^	2.93	*	0.08	0.86	0.94
Σ Med chain	52.3 ^a^	55.8 ^ab^	1.24	52.6 ^a^	57.4 ^ab^	1.75	58.7 ^b^	60.6 ^b^	2.47	*	*	0.82	0.80
Σ Branched	2.02 ^a^	1.88 ^a^	0.060	2.94 ^a^	1.97 ^a^	0.084	1.75 ^ab^	1.71 ^ab^	0.119	0.27	0.08	0.79	0.54
Σ Trans	3.58	3.49	0.234	3.77	3.17	0.331	2.57	2.67	0.468	0.42	0.06	0.13	0.59
Σ Saturated	69.8 ^b^	72.5 ^a^	1.13	69.8 ^b^	73.2 ^a^	1.60	75.3 ^a^	75.8 ^a^	2.26	*	0.06	0.84	0.75
Σ MUFA	22.0 ^b^	19.1 ^a^	1.11	21.7 ^b^	18.8 ^a^	1.56	17.1 ^ab^	16.0 ^a^	2.21	*	0.09	0.75	0.87
Σ PUFA	2.35	2.35	0.093	2.56	2.19	0.132	2.10	2.07	0.186	0.29	0.19	0.38	0.30
C4	3.63 ^a^	3.37 ^b^	0.104	3.66 ^a^	3.10 ^b^	0.147	3.50 ^a^	3.42 ^ab^	0.208	**	0.61	*	0.33
C6	2.26 ^b^	2.43 ^a^	0.038	2.33 ^a^	2.28 ^a^	0.054	2.33 ^a^	2.35 ^a^	0.076	*	0.73	0.38	0.06
C8	1.38 ^b^	1.61 ^a^	0.044	1.44 ^a^	1.42 ^a^	0.062	1.43 ^a^	1.46 ^a^	0.087	**	0.50	0.53	0.07
C10	3.47 ^b^	4.38 ^a^	0.177	3.63 ^a^	3.89 ^a^	0.251	3.93 ^a^	4.33^a^	0.355	**	0.50	0.56	0.32
C10:1	0.26 ^b^	0.32 ^a^	0.013	0.28 ^a^	0.28 ^a^	0.019	0.28 ^a^	0.28 ^a^	0.027	*	0.79	0.53	0.15
C12	4.32 ^b^	5.49 ^a^	0.285	4.25 ^a^	4.92 ^a^	0.404	5.01 ^a^	5.67 ^a^	0.571	**	0.32	0.32	0.72
C14	12.3 ^a^	13.5 ^b^	0.36	12.4 ^a^	13.7 ^b^	0.51	14.0 ^b^	14.4 ^bc^	0.73	**	0.09	0.36	0.78
C14:1 *cis* 9	0.65 ^a^	0.90 ^b^	0.063	0.76 ^a^	0.84 ^b^	0.089	0.59 ^a^	0.86 ^b^	0.126	*	0.73	0.18	0.37
C16:0	29.7 ^a^	30.7 ^a^	0.71	30.0 ^a^	32.7 ^a^	0.98	33.9 ^b^	34.4 ^b^	1.38	0.08	**	0.84	0.57
C16:1	1.11	1.12	0.066	1.10	1.11	0.093	0.99	1.13	0.132	0.69	0.81	0.06	0.76
C18:0	9.90 ^a^	8.34 ^b^	0.424	9.62 ^a^	8.56 ^a^	0.599	8.57 ^a^	7.00 ^a^	0.847	**	0.14	0.77	0.89
C18:1 *trans* 9	0.14	0.13	0.004	0.13	0.12	0.006	0.12	0.11	0.008	0.06	0.07	0.06	0.90
C18:1 *trans* 11	3.44	3.36	0.232	3.64	3.05	0.328	2.45	2.55	0.464	0.43	0.07	0.14	0.60
C18:1 *cis* 9	16.3 ^b^	13.5 ^a^	1.03	15.8 ^a^	13.5 ^a^	1.46	12.5 ^a^	11.2 ^a^	2.07	*	0.20	0.56	0.90
C18:1 *cis* 11	0.60 ^b^	0.52 ^a^	0.025	0.57 ^a^	0.52 ^a^	0.025	0.55 ^a^	0.51 ^a^	0.050	*	0.80	0.34	0.79
C18:2 *cis* 9, 12	0.49	0.46	0.018	0.51	0.46	0.026	0.53	0.54	0.036	0.09	0.17	0.66	0.63
C18:3 *cis* 9, 12, 15	0.69	0.66	0.033	0.76	0.67	0.047	0.79	0.69	0.067	0.17	0.39	0.98	0.66
CLA ^3^	1.18 ^a^	1.23 ^a^	0.080	1.29 ^a^	1.06 ^a^	0.113	0.80 ^b^	0.85 ^b^	0.160	0.79	*	0.26	0.35

^a–b^ means in the same row with different superscripts differ (*p* < 0.05). *p* < 0.05 *; *p <* 0.01**; *p* < 0.001 ***. ^1^ Stage one: transition to FB allocation days 1–12; stage two: days 13–17; stage three: days 18–20. ^2^ SE: standard error of estimated marginal means. ^3^ CLA: conjugated linoleic acid *cis* 9 *trans* 11. ^4^ Diet by stage interaction.

**Table 4:** Daily mean, maximum and nadir rumen pH and duration (minutes) where pH was below 6.0, 5.8 and 5.6 during the three stages of adaptation to either fodder beet bulb and herbage (FBH) or herbage-only (HO) diets.

Adaptation Stage ^1^	Stage One		SE ^2^	Stage Two		SE	Stage Three		SE	*p*-Value				
	HO	FBH		HO	FBH		HO	FBH		Diet	Stage	Period	Cow	D × S^3^
Nadir pH	5.62 ^a^	5.57 ^a^	0.04	5.64 ^a^	5.47 ^b^	0.04	5.71 ^a^	5.48 ^b^	0.05	***	0.41	***	***	*
Max pH	6.35 ^b^	6.43 ^a^	0.02	6.46 ^c^	6.48 ^c^	0.03	6.46 ^c^	6.55 ^d^	0.04	0.15	***	***	***	**
Mean pH	6.01 ^b^	6.03 ^b^	0.002	6.09 ^d^	6.04 ^c^	0.003	6.16 ^f^	6.08 ^e^	0.004	***	***	***	***	***
pH < 6.0	516 ^b^	475 ^a^	2.8	392 ^d^	441 ^c^	3.2	349 ^f^	369 ^e^	3.7	***	***	***	***	***
pH < 5.8	142 ^b^	126 ^a^	1.5	118 ^d^	134 ^c^	1.7	99 ^f^	108 ^e^	1.8	***	***	***	***	***
pH < 5.6	12.2 ^b^	20.9 ^a^	0.7	10.9 ^d^	30.0 ^c^	0.9	10.9 ^d^	20.8 ^a^	0.8	***	***	***	***	***

^a–f^ means in the same row with different superscripts differ (*p* < 0.05). *p* < 0.05 *; *p* < 0.01**; *p* < 0.001 ***. ^1^ Stage one: transition to FB allocation days 1–12; stage two: days 13–17; stage three: days 18–20. ^2^ SE: standard error of estimated marginal means. ^3^ Diet by adaptation stage interaction.

**Table 5:** Rumen concentration of volatile fatty acids (VFA: mmol/L) and lactate (mol/L) during dietary adaptation to either herbage and fodder beet bulb (FBH) or herbage-only (HO) diets.

Adaptation Stage ^1^	1		SE ^2^	2		SE	3		SE	*p*-Value				
Diet	HO	FBH		HO	FBH		HO	FBH		Diet	Day	Time	Period	D × D × T ^5^
Lactate (mol/L)	0.94 ^a^	2.60 ^a^	1.851	3.20 ^a^	84.7^c^	27.0	0.70 ^a^	4.35 ^b^	2.212	**	***	***	**	***
Ammonia (mmol/L)	4.58 ^a^	4.04 ^a^	0.368	3.42 ^c^	2.28 ^b^	0.270	3.59 ^c^	1.53 ^b^	0.231	**	***	***	0.07	*
tVFA ^3^ (mmol/L)	117 ^a^	118 ^a^	2.5	119 ^a^	103 ^b^	2.6	118 ^a^	107 ^b^	2.6	***	*	***	*	0.81
Molar Proportions														
Acetate	60.8 ^a^	59 ^b^	0.31	60.5 ^a^	55.0 ^b^	0.28	63.3 ^a^	56.5 ^b^	0.29	***	***	***	**	***
Propionate	21.1 ^a^	21.5 ^a^	0.22	21.2 ^a^	20.6 ^b^	0.22	20.0 ^b^	19.8 ^c^	0.21	***	*	***	0.64	*
Iso -butyrate	0.73 ^a^	0.78 ^b^	0.013	0.72 ^a^	0.50 ^c^	0.011	0.71 ^a^	0.55 ^c^	0.011	***	***	***	***	***
Butyrate	13.3 ^a^	14.2 ^b^	0.20	14.0 ^b^	17.6 ^d^	0.21	12.7 ^e^	15.4 ^c^	0.19	***	***	***	0.42	**
Iso-valerate	1.06 ^a^	1.13 ^ab^	0.027	1.05 ^a^	0.68 ^b^	0.020	1.05 ^a^	0.59 ^b^	0.019	***	***	***	0.48	***
Valerate	1.46 ^a^	1.32 ^ab^	0.050	1.35 ^a^	2.38 ^b^	0.070	1.43 ^a^	1.25 ^c^	0.073	***	***	***	0.71	1.00
Hexanoic	0.55 ^a^	0.60 ^a^	0.019	0.53 ^a^	0.82 ^b^	0.023	0.50 ^a^	0.88 ^c^	0.023	***	***	***	*	0.16
A + B/P ^4^	3.54 ^a^	3.47 ^a^	0.058	3.49 ^a^	3.62 ^ab^	0.062	3.8 1^b^	3.81 ^b^	0.067	0.66	***	***	**	0.06

^a–e^ means in the same row with different superscripts differ (*p* < 0.05). *p* < 0.05 *; *p* < 0.01 **; *p* < 0.001 ***^. 1^ Stage one: transition to FB allocation days 1–12; stage two: days 13–17; stage three: days 18–20. ^2^ SE: standard error of estimated marginal means. ^3^ total VFA. ^4^ Acetate + butyrate: propionate ratio. ^5^ Diet, day and time interaction.

**Table 6:** Plasma concentration (μmol/L) of amino acids and plasma nonesterified fatty acid (NEFA mmol/L) during dietary adaptation to either a fodder beet bulbs and herbage (FBH) or herbage-only (HO) diets.

Adaptation Stage ^1^	1		SE ^2^	2		SE	3		SE	*p*-Value			
Diet	HO	FBH		HO	FBH		HO	FBH		Diet	Stage	Period	D × S ^3^
Glutamic acid (μmol/L)	73.7 ^a^	74.8 ^a^	2.57	80.0 ^ab^	87.0 ^b^	2.57	78.0 ^b^	85.9 ^b^	2.66	*	***	1.00	0.35
Aspartate	8.11 ^a^	9.72 ^a^	0.765	11.26 ^b^	11.33 ^b^	0.765	10.69 ^b^	12.28 ^b^	0.793	0.10	**	**	0.52
Cysteine	99.3	99.5	3.99	106	103	3.99	105	101	4.13	0.47	0.42	*	0.85
Asparagine	8.11^a^	9.72 ^a^	0.765	11.26 ^b^	11.33 ^b^	0.765	10.69 ^b^	12.28 ^b^	0.793	0.10	**	**	0.52
Serine	43.0 ^a^	47.9 ^a^	3.29	44.1 ^a^	43.5 ^a^	3.29	42.0 ^a^	35.6 ^b^	3.34	0.63	**	0.18	*
Glutamine	103	94.8	9.04	99.6	103	9.04	93.7	86.0	9.33	0.50	0.36	0.36	0.73
Histidine	27.0 ^a^	28.0 ^a^	1.30	26.6 ^b^	24.1 ^b^	1.30	21.3 ^c^	19.3 ^c^	1.34	0.24	***	***	0.28
Glycine	97.4 ^a^	112 ^a^	13.9	79.4 ^a^	182 ^c^	13.9	86.6 ^a^	110 ^b^	14.4	***	*	0.22	**
Threonine	98.3 ^b^	103 ^b^	8.70	103 ^b^	120 ^bc^	8.70	91.0 ^a^	89.5 ^a^	8.94	0.29	*	0.27	0.42
Arginine	49.9	53.2	4.51	54.7	54.2	4.51	54.8	48.5	4.68	0.73	0.77	**	0.57
Alanine	122 ^a^	138 ^a^	12.9	162 ^b^	153 ^b^	12.9	131 ^a^	114 ^a^	13.4	0.60	**	**	0.23
Taurine	23.3 ^a^	23.8 ^a^	1.24	24.5 ^a^	30.4 ^b^	1.20	23.8 ^a^	26.7 ^a^	1.28	**	**	***	0.07
Tyrosine	42.2 ^a^	44.2 ^a^	4.44	47.4 ^a^	43.2 ^a^	4.44	37.0 ^b^	33.6 ^b^	4.60	0.52	0.06	0.81	0.73
Valine	148	167	13.8	158	168	13.8	147	133	14.3	0.66	0.23	0.37	0.46
Methionine	18.7 ^a^	19.1 ^a^	1.70	20.0 ^a^	21.4 ^a^	1.70	15.3 ^b^	14.9 ^b^	1.76	0.79	**	0.92	0.88
Tryptophan	42.2 ^a^	44.4 ^a^	4.44	47.4 ^a^	43.2 ^a^	4.44	37.0 ^b^	33.6 ^b^	5.60	0.52	0.06	0.81	0.73
Phenylalanine	41.3 ^a^	44.3 ^a^	3.83	46.5 ^a^	43.8 ^a^	3.83	38.2 ^ab^	32.6 ^ab^	3.97	0.53	0.04	0.52	0.54
Isoleucine	93.6	103	9.76	96.1	128	9.76	91.8	93.8	8.9	0.09	0.15	0.86	0.31
Lysine	31.9	41.9	6.36	35.8	39.6	6.36	24.4	20.5	6.60	0.58	*	0.28	0.57
Leucine	93.5	104	10.2	95.4	93.2	10.2	86.8	71.4	10.6	0.74	0.15	0.81	0.47
Proline	71.0	71.8	5.11	73.4	77.2	5.11	75.3	73.9	5.25	0.75	0.61	0.49	0.82
NEFA (mmol/L)	0.08 ^a^	0.08 ^a^	0.0064	0.08 ^a^	0.05 ^b^	0.0064	0.07 ^a^	0.06 ^ab^	0.0066	*	*	0.60	0.09

^a–f^ means in the same row with different superscripts differ (*p* < 0.05). *p* < 0.05 *; *p* < 0.01**; *p* < 0.001 ***. ^1^ Stage one: transition to FB allocation days 1–12; stage two: days 13–17; stage three: days 18–20. ^2^ SE: standard error. ^3^ Diet by adaptation stage interaction.
